# Supplementary material for: Causal Relationship between Aging and Anorexia Nervosa: A White-Matter-Microstructure-Mediated Mendelian Randomization Analysis
Source: Biomedicines. 2024 Aug 16;12(8):1874. doi: 10.3390/biomedicines12081874 (PMC11351342; doi:10.3390/biomedicines12081874)
Supplement: Supplementary file 1 [file biomedicines-12-01874-s001.zip › Supplementary Figure/Figure S4/Singe SNP plot of MR analysis for TL to ubm-b-1901.pdf]

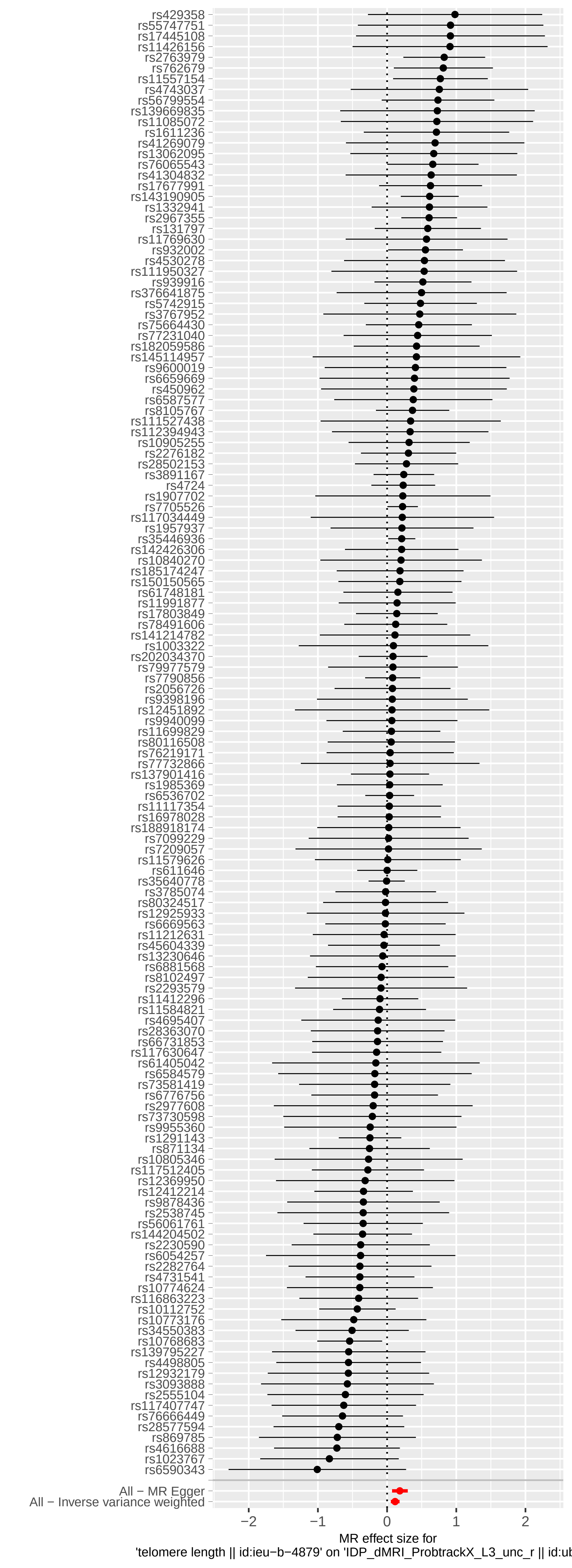
rs429358
rs55747751
rs17445108
rs11426156
rs2763979
rs762679
rs11557154
rs4743037
rs56799554
rs139669835
rs11085072
rs1611236
rs41269079
rs13062095
rs76065543
rs41304832
rs17677991
rs143190905
rs1332941
rs2967355
rs131797
rs11769630
rs932002
rs4530278
rs111950327
rs939916
rs376641875
rs5742915
rs3767952
rs75664430
rs77231040
rs182059586
rs145114957
rs9600019
rs6659669
rs450962
rs6587577
rs8105767
rs111527438
rs112394943
rs10905255
rs2276182
rs28502153
rs3891167
rs4724
rs1907702
rs7705526
rs117034449
rs1957937
rs35446936
rs142426306
rs10840270
rs185174247
rs150150565
rs61748181
rs11991877
rs17803849
rs78491606
rs141214782
rs1003322
rs202034370
rs79977579
rs7790856
rs2056726
rs9398196
rs12451892
rs9940099
rs11699829
rs80116508
rs76219171
rs77732866
rs137901416
rs1985369
rs6536702
rs11117354
rs16978028
rs188918174
rs7099229
rs7209057
rs11579626
rs611646
rs35640778
rs3785074
rs80324517
rs12925933
rs6669563
rs11212631
rs45604339
rs13230646
rs6881568
rs8102497
rs2293579
rs11412296
rs11584821
rs4695407
rs28363070
rs66731853
rs117630647
rs61405042
rs6584579
rs73581419
rs6776756
rs2977608
rs73730598
rs9955360
rs1291143
rs871134
rs10805346
rs117512405
rs12369950
rs12412214
rs9878436
rs2538745
rs56061761
rs144204502
rs2230590
rs6054257
rs2282764
rs4731541
rs10774624
rs116863223
rs10112752
rs10773176
rs34550383
rs10768683
rs139795227
rs4498805
rs12932179
rs3093888
rs2555104
rs117407747
rs76666449
rs28577594
rs869785
rs4616688
rs1023767
rs6590343
All − MR Egger
All − Inverse variance weighted
−2
−1
0
1
2
MR effect size for
'telomere length || id:ieu−b−4879' on 'IDP_dMRI_ProbtrackX_L3_unc_r || id:ub
